# Biosynthesis of cyanogenic glucosides in *Phaseolus lunatus* and the evolution of oxime‐based defenses

**DOI:** 10.1002/pld3.244

**Published:** 2020-08-04

**Authors:** Daniela Lai, Alexandra B. Maimann, Eliana Macea, César H. Ocampo, Gustavo Cardona, Martina Pičmanová, Behrooz Darbani, Carl Erik Olsen, Daniel Debouck, Bodo Raatz, Birger Lindberg Møller, Fred Rook

**Affiliations:** ^1^ Plant Biochemistry Laboratory Department of Plant and Environmental Sciences University of Copenhagen Frederiksberg Denmark; ^2^ VILLUM Center for Plant Plasticity University of Copenhagen Frederiksberg Denmark; ^3^ International Center for Tropical Agriculture Cali Colombia; ^4^ Present address: The Novo Nordisk Foundation Center for Biosustainability Technical University of Denmark Lyngby Denmark

**Keywords:** chemical defense, cyanogenic glucosides, cytochrome P450, *Phaseolus lunatus*

## Abstract

Lima bean, *Phaseolus lunatus*, is a crop legume that produces the cyanogenic glucosides linamarin and lotaustralin. In the legumes *Lotus japonicus* and *Trifolium repens*, the biosynthesis of these two α‐hydroxynitrile glucosides involves cytochrome P450 enzymes of the CYP79 and CYP736 families and a UDP‐glucosyltransferase. Here, we identify CYP79D71 as the first enzyme of the pathway in *P. lunatus*, producing oximes from valine and isoleucine. A second CYP79 family member, CYP79D72, was shown to catalyze the formation of leucine‐derived oximes, which act as volatile defense compounds in *Phaseolus* spp. The organization of the biosynthetic genes for cyanogenic glucosides in a gene cluster aided their identification in *L. japonicus*. In the available genome sequence of *P. vulgaris*, the gene orthologous to *CYP79D71* is adjacent to a member of the *CYP83* family. Although *P. vulgaris* is not cyanogenic, it does produce oximes as volatile defense compounds. We cloned the genes encoding two CYP83s (CYP83E46 and CYP83E47) and a UDP‐glucosyltransferase (UGT85K31) from *P. lunatus*, and these genes combined form a complete biosynthetic pathway for linamarin and lotaustralin in Lima bean. Within the genus *Phaseolus*, the occurrence of linamarin and lotaustralin as functional chemical defense compounds appears restricted to species belonging to the closely related Polystachios and Lunatus groups. A preexisting ability to produce volatile oximes and nitriles likely facilitated evolution of cyanogenesis within the *Phaseolus* genus.

## 1 INTRODUCTION

The legume family contains a large number of economically important crops that are high in protein content. Within the genus *Phaseolus*, multiple species were domesticated, and in the cases of the common bean (*P. vulgaris*) and Lima bean (*P. lunatus*), domestication occurred twice independently (Schmutz et al., 2014; Serrano‐Serrano, Andueza‐Noh, Martínez‐Castillo, Debouck, & Chacón, 2012). The full potential of *P. lunatus* as a legume crop for food or feed has been hampered by the release of toxic hydrogen cyanide (HCN) from damaged leaves and seeds (Baudoin, Barthelemy, & Ndungo, 1991), whereas *P. vulgaris* is not known to be a cyanogenic species. Within the genus *Phaseolus*, cyanogenesis has only been reported for five species cross‐compatible with *P. lunatus* (Baudoin et al., 1991).

Cyanogenesis is characterized by the release of HCN from damaged tissues and is an example of a two‐component plant chemical defense system (Gleadow & Møller, 2014). Cyanogenic glucosides are synthesized from specific amino acids and the predominant cyanogenic glucosides in *P. lunatus* are linamarin, which is derived from valine, and lotaustralin, derived from isoleucine. In plant tissues, cyanogenic glucosides are stored separately from the β‐glucosidase enzymes triggering HCN release, and the two components only come into contact following tissue disruption, such as caused by feeding insects (Frehner & Conn, 1987; Lai et al., 2014). The hydrolysis of cyanogenic glucosides releases their unstable α‐hydroxynitrile aglycone, which dissociates with the formation of toxic HCN.

The first biosynthetic pathway for a cyanogenic glucoside to be elucidated was that of dhurrin in the monocot *Sorghum bicolor*. Dhurrin is synthesized from tyrosine which is converted to an oxime, *E‐p*‐hydroxyphenylacetaldoxime, by the action of the cytochrome P450 enzyme CYP79A1 (Koch, Sibbesen, Halkier, Svendsen, & Møller, 1995). A second cytochrome P450, CYP71E1, catalyzes conversion of the oxime to *p*‐hydroxymandelonitrile, which is glucosylated by the UDP‐glucosyltransferase UGT85B1 to yield dhurrin (Bak, Kahn, Nielsen, Møller, & Halkier, 1998; Jones, Møller, & Høj, 1999; Laursen et al., 2016). Similar biosynthetic pathways involving oximes and α‐hydroxynitrile producing cytochrome P450 enzymes, have been shown to exist for the synthesis of specific cyanogenic glucosides in cassava (*Manihot esculenta*), the model legume *Lotus japonicus*, white clover (*Trifolium repens*), Japanese apricot (*Prunus mume*), almond (*Prunus dulcis*), and sugar gum (*Eucalyptus cladocalyx*) (Andersen, Busk, Svendsen, & Møller, 2000; Forslund et al., 2004; Hansen et al., 2018; Olsen & Small, 2018; Sánchez‐Pérez et al., 2019; Takos et al., 2011; Thodberg et al., 2018; Yamaguchi, Yamamoto, & Asano, 2014). Whereas the first enzyme is invariably a cytochrome P450 of the CYP79 family converting a specific amino acid into an oxime, the α‐hydroxynitrile producing second enzymatic step can be catalyzed by members of the CYP71, CYP736, or CYP706 families (Bak et al., 1998; Hansen et al., 2018; Takos et al., 2011; Yamaguchi et al., 2014). Although cyanogenesis was initially thought of as an “ancient” chemical defense trait because of its widespread occurrence in over 130 plant families (Bak et al., 2006), we more recently proposed that cyanogenesis repeatedly evolved independently in several plant lineages by the recruitment of members from the same or similar gene families (Takos et al., 2011). This type of “repeated” or convergent evolution is surprisingly common in plant specialized metabolism (Pichersky & Lewinsohn, 2011).

Genome analysis has contributed to the elucidation of the biosynthetic pathway for cyanogenic glucosides in some species. Analysis of the *L. japonicus* genome revealed that the biosynthetic genes for linamarin and lotaustralin are organized in a biosynthetic gene cluster and helped identify CYP736A2 as responsible for the second enzymatic step (Takos et al., 2011). We also reported the existence of biosynthetic gene clusters for cyanogenic glucosides in the genomes of cassava and sorghum, of which the latter was shown to contain additionally a vacuolar MATE‐type transporter for dhurrin (Darbani et al., 2016). A gene cluster in barley (*Hordeum vulgare*) contains the CYP79 and CYP71 genes that encode the enzymes for the production of five leucine‐derived α‐, β‐, and γ‐hydroxynitrile glucosides, including the cyanogenic glucoside epiheterodendrin (Knoch, Motawie, Olsen, Møller, & Lyngkjær, 2016). Biosynthetic gene clusters are being reported for an increasing number of plant specialized defense metabolites, and may form by selection for reduced recombination between interacting alleles for traits that are under balancing selection, thus promoting the co‐inheritance of functional pathways (Boycheva, Daviet, Wolfender, & Fitzpatrick, 2014; Takos & Rook, 2012).

**FIGURE 1:**
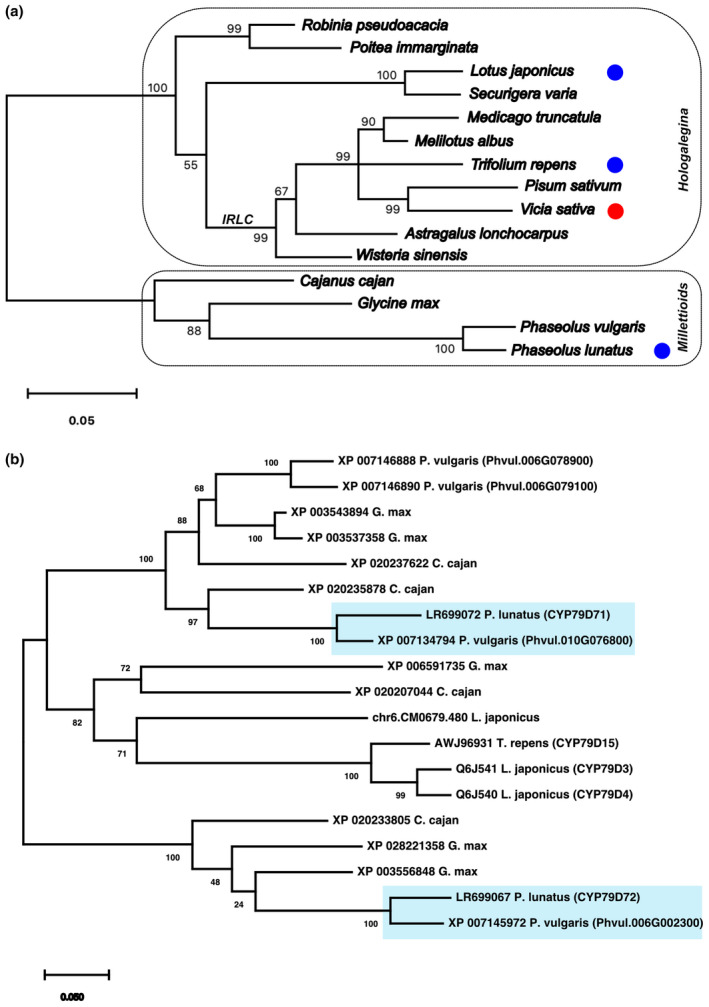
(a) Simplified phylogenetic tree of selected species belonging to the NPAAA‐clade within the legume family. It shows the Millettioid subclade containing the genus *Phaseolus*, and the Hologalegina subclade containing the genera *Lotus*, *Trifolium,* and *Vicia*. The presence of cyanogenic glucosides in a species is indicated by circles: linamarin/lotaustralin (blue) and prunasin/vicianin (red). The age of the Hologalegina diversification is estimated at about 50 million years ago, and that of the Millettioid clade at 45 million years ago (Lavin, Herendeen, & Wojciechowski, 2005). IRLC indicates the inverted‐repeat‐lacking clade. The phylogenetic analysis is based on chloroplast matK amino acid sequences. For a comprehensive overview of legume phylogeny see Wojciechowski et al. (2004). (b) A phylogenetic analysis of legume cytochrome P450 enzymes of the CYP79D‐subfamily. CYP79 protein sequences from six legume species are included: *P. lunatus* (Lima bean), *P. vulgaris* (common bean), *Glycine max* (soybean), *Cajanus cajan* (pigeon pea), *Trifolium repens* (white clover), and *Lotus japonicus*. Names represent GenBank accession numbers and/or with chromosomal locations and assigned names in parentheses. Phylogenetic analyses were performed with the Maximum Likelihood method and the Jones‐Taylor‐Thornton (JTT) matrix‐based model for amino acid sequences, using the MEGA X software. Positions containing gaps were eliminated and bootstrap values (1000x) are indicated at the branch points. Branch lengths are measured in the number of substitutions per site

Within the legumes, linamarin and lotaustralin are the predominant cyanogenic glucosides found in *L. japonicus*, white clover (*T. repens*), and *P. lunatus*, whereas *Vicia* spp. produce the phenylalanine‐derived cyanogenic glucosides prunasin and vicianin (Ahn, Saino, Mizutani, Shimizu, & Sakata, 2007; Aouida et al., 2019). Recently, Olsen and Small (2018) reported that all the biosynthetic genes for the two cyanogenic glucosides in white clover were orthologous to the ones in *L. japonicus* and also organized in a biosynthetic gene cluster. Within the legume subfamily Papilionoideae, the genera *Lotus* and *Trifolium* are part of the Hologalegina clade, whereas the genus *Phaseolus* is more distantly related and part of the phaseoloid/millettioid group (Figure 1a; Doyle & Luckow, 2003; Wojciechowski, Lavin, & Sanderson, 2004). Determining if cyanogenesis in the genus *Phaseolus* evolved independently or not from its occurrence in the Hologalegina clade, will, therefore, provide further insights into the evolutionary dynamics of this plant chemical defense system.

Besides being an important legume crop, *P. lunatus* is also extensively used as an experimental plant in chemical ecology to study variations in cyanogenesis and their effects on herbivore behavior, and the various trade‐offs between defense traits (Ballhorn, Kautz, Heil, & Hegeman, 2009; Ballhorn et al., 2011). For example, cyanogenesis as a direct defense was negatively correlated with the emission of volatile organic compounds (VOCs) as an indirect defense against herbivores (Ballhorn, Kautz, Lion, & Heil, 2008). Therefore, identifying the genes encoding the biosynthetic pathway for cyanogenic glucosides in *P. lunatus* benefits breeding efforts, supports ecological research, and allows a comparative analysis of cyanogenesis and its evolution in a third legume species.

## 2 RESULTS

### 2.1 Cloning of two CYP79 genes from *P. lunatus* using sequence homology

The first step in biosynthetic pathways for cyanogenic glucosides in seed plants is the conversion of an amino acid into an oxime by a cytochrome P450 of the CYP79 family. To identify CYP79 candidate genes from *P. lunatus* involved in cyanogenic glucoside biosynthesis, we used a PCR‐based approach using degenerate primers. Four conserved amino acid motifs present in legume CYP79s were selected that distinguish these enzymes from other cytochrome P450 families. The four amino acid motifs were GNLPEMLAN, MKEMNTEIACIRL, LAEMINQPELL, and LGTTMT (V/I) (M/I) LFAR. The corresponding DNA sequences were obtained from *L. japonicus* (*CYP79D3*), *T. repens* (*CYP79D15*), and *P. vulgaris* (*CYP79D39*) and used in the design of degenerate primers (Table S1). Using the primers in different combinations, four separate PCR products of expected lengths were amplified from *P. lunatus* cDNA obtained from young leaves, a tissue with a high level of cyanogenic glucoside production (Figure S1). Cloning and sequencing of the PCR fragments revealed that they represented fragments of two distinct gene sequences. Subsequent use of gene specific primers (Table S1) in 5′‐ and 3′‐RACE‐PCR procedures provided the full‐length cDNA sequences for both genes. Phylogenetic analysis of their amino acid sequence placed both cytochrome P450 enzymes in the CYP79D‐subfamily (Figure 1b), and they were assigned the names CYP79D71 and CYP79D72 by the cytochrome P450 nomenclature committee (Nelson, 2009). For clarity, in this paper prefixes are used to indicate the plant species, for example, *Pl*CYP79D71.

Although members of the same sub‐family, *Pl*CYP79D71 and *Pl*CYP79D72 only shared 65% amino acid identity between them. Both genes have likely orthologs in the available *P. vulgaris* genome sequence (Schmutz et al., 2014). *Pl*CYP79D71 showed 91% amino acid sequence identity with the enzyme encoded by *Phvul.010G076800*, whereas *Pl*CYP79D72 shared 92% amino acid sequence identity with the protein encoded by *Phvul.006G002300*. Of the two, *Pl*CYP79D71 was most closely related to *Lj*CYP79D3 and *Lj*CYP79D4 from *L. japonicus* and *Tr*CYP79D15 from *T. repens*, all three of which produce valine and isoleucine‐derived oximes in the biosynthesis of the cyanogenic glucosides linamarin and lotaustralin.

**FIGURE 2:**
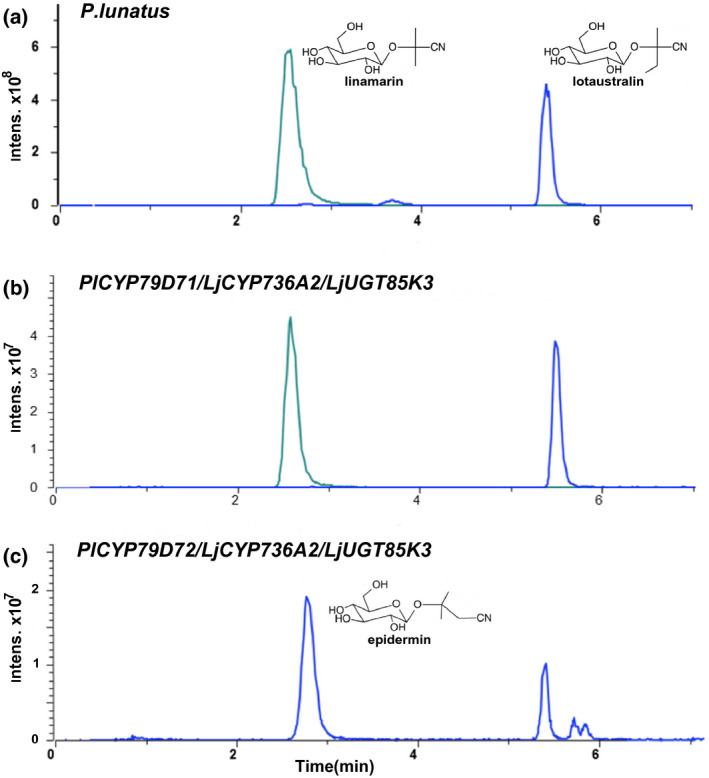
Extracted ion chromatograms of linamarin and lotaustralin production in the leaves of *P. lunatus* and following co‐expression of *PlCYP79D71* in *N. benthamiana*. (a) Metabolic profile of a young *P. lunatus* leaf. (b) Metabolic profile of infiltrated tobacco leaves co‐expressing *CYP79D71* from *P. lunatus*, with *CYP736A2* and *UGT85K3* from *L. japonicus*. (c) Metabolic profile of infiltrated tobacco leaves co‐expressing *CYP79D72* from *P. lunatus*, with *CYP736A2* and *UGT85K3* from *L. japonicus*. Extracted ion peaks are for sodium adducts: linamarin (m/z 270, cyan), lotaustralin (m/z 284, blue), epidermin (at 2.7 min in panel c, m/z 284, blue)

To evaluate both enzymes for their ability to function in the biosynthesis of linamarin and lotaustralin, the cyanogenic glucosides found in *P. lunatus* (Figure 2a), both genes were transiently expressed in leaves of *Nicotiana benthamiana* using Agrobacterium infiltration. Separately, the two *CYP79* genes from *P. lunatus* were co‐expressed with *LjCYP736A2* and *LjUGT85K3*, encoding the second and third enzymes of the biosynthetic pathway for the cyanogenic glucosides linamarin and lotaustralin in *L. japonicus* (Takos et al., 2011). Chemical analysis of the infiltrated leaves using liquid chromatography–mass spectrometry (LC–MS) showed that the combination of *PlCYP79D71/LjCYP736A2/LjUGT85K3* resulted in the production of linamarin and lotaustralin (Figure 2b). Some lotaustralin was present in the samples extracted from tobacco leaves expressing the gene combination *PlCYP79D72/LjCYP736A2/LjUGT85K3* (Figure 2c), but here the main product was a compound with a mass‐to‐charge ratio (*m/z*) of 284 eluting at 2.7 min Based on these characteristics, its MS2 fragmentation pattern, and comparison with a standard, this compound was identified as the β‐hydroxynitrile glucoside epidermin (Figure 2c, Figure S2). A minor compound with *m/z* 284 at 5.6 min was identified as the cyanogenic glucoside epiheterodendrin. Both these hydroxynitrile glucosides are derived from leucine and occur naturally in barley (*Hordeum vulgare*) (Knoch et al., 2016), but have not been reported in *P. lunatus*. We, therefore, propose that *Pl*CYP79D72 prefers leucine as its main substrate, whereas *Pl*CYP79D71 is the enzyme likely responsible for producing the valine and isoleucine‐derived oximes in the synthesis of the cyanogenic glucosides linamarin and lotaustralin in *P. lunatus*.

### 2.2 A small gene cluster in *P. vulgaris* associates CYP79D71 with CYP83s

In cassava (*M. esculenta*), sorghum (*S. bicolor*) and *L. japonicus*, the biosynthetic pathways for cyanogenic glucosides are organized in genomic gene clusters (Takos et al., 2011). Such an organization greatly facilitates gene discovery, but requires the availability of a genome sequence. A draft genome sequence is available for *P. vulgaris* (Schmutz et al., 2014) and although this species is non‐cyanogenic, reports of the occurrence of low amounts of linamarin exist (Johne, 1991). In addition, *P. vulgaris* produces valine, leucine and isoleucine‐derived oximes as volatile defense compounds (Wei, Zhu, & Kang, 2006). We, therefore, considered the possible existence of synteny between the *P. vulgaris* and *P. lunatus* genomes. In the *P. vulgaris genome, Phvul.10G076800,* the gene orthologous to *PlCYP79D71*, is localized on chromosome 10. This genomic region additionally contains cytochrome P450 genes belonging to the CYP83 gene family, the sister‐family to the CYP71s (Nelson & Werck‐Reichhart, 2011). Oxime metabolizing CYP83s were previously reported in the biosynthesis of glucosinolates, which made these CYP83s plausible candidate enzymes for the oxime to nitrile conversion in the production of volatiles and cyanogenic glucosides (Halkier & Gershenzon, 2006; Naur et al., 2003). *Phvul.010G076700* encodes a member of the CYP83‐family and is positioned immediately upstream of *Phvul.10G076800* in both the original draft (v1.0) and the current release of the *P. vulgaris* genome (v2.1, https://phytozome.jgi.doe.gov/pz/portal.html). A second functional CYP83 is encoded by *Phvul.010G077000*, showing 60% amino acid identity with the CYP83 encoded by *Phvul.010G076700*, but its relative position to the other two genes is more distant in version 2.1 of the *P. vulgaris* genome.

Based on the DNA sequences of *Phvul.010G076700* and *Phvul.010G077000* we designed degenerate primers (Table S1) to isolate *CYP83* genes from *P. lunatus* cDNA. Full length cDNA clones of two distinct *CYP83* genes expressed in young leaves of *P. lunatus* were obtained, and the encoded proteins were assigned the names CYP83E46 and CYP83E4*7* (Nelson, 2009). Both *P. lunatus* genes were most closely related to *Phvul.010G076700*, showing, respectively, 90% and 86% amino acid sequence identity. Their similarity to the enzyme encoded by *Phvul.010G077000* was much lower at around 60% amino acid sequence identity. CYP83E46 and CYP83E47 shared 82% amino acid sequence identity between them, and as the *P. vulgaris* genome only seems to contain a single gene copy, they potentially are diverging paralogs.

### 2.3 CYP79D71, CYP83E46/47, and UGT85K31 constitute a functional biosynthetic pathway for linamarin and lotaustralin in *P. lunatus*

The genomic region in *P. vulgaris* surrounding the CYP79 and CYP83 genes, does not contain an UDP‐glucosyltransferase encoding gene. Known UDP‐glucosyltransferases involved in cyanogenic monoglucoside biosynthesis belong to the UGT85 family. The UGT85 encoding genes in the *P. vulgaris* genome that show most sequence similarity with *Lj*UGT85K3 from *L. japonicus* are *Phvul.006G017500* and *Phvul.006G017600*. Using degenerate primers based on these gene sequences (Table S1), we isolated a single partial UGT85 gene sequence from leaf cDNA. Using 3′ and 5′ RACE, a full‐length sequence was subsequently obtained. The encoded protein showed 89% amino acid sequence identity with Phvul.006G017500 and 69% with *Lj*UGT85K3, and was assigned the name UGT85K31.

**FIGURE 3:**
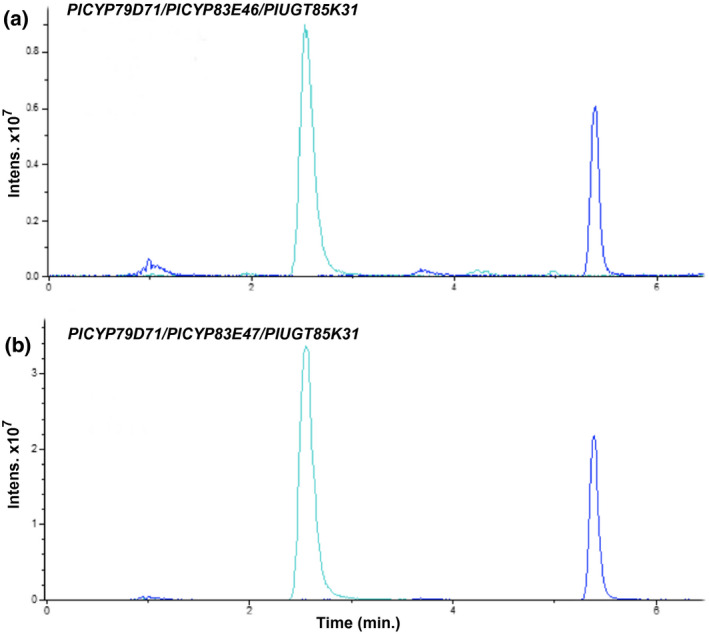
The biosynthetic pathway for linamarin and lotaustralin in *P. lunatus* consists of CYP79D71, CYP83E46/CYP83E47, and UGT85K31. Metabolic profiles of *N. benthamiana* leaves co‐infiltrated with the *P. lunatus* gene combinations: (a) *CYP79D71*, *CYP83E46*, and *UGT85K31*. (b) *CYP79D71*, *CYP83E47*, and *UGT85K31*. Extracted ion peaks are for sodium adducts: linamarin (m/z 270, cyan) and lotaustralin (m/z 284, blue)

**FIGURE 4:**
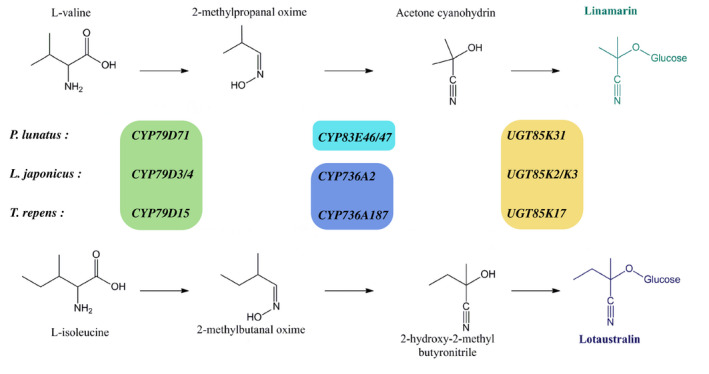
Schematic drawing of the biosynthetic pathway for linamarin and lotaustralin in three legume species. Members of the CYP79 family convert valine and isoleucine into their corresponding oximes. The oxime metabolizing enzyme is a CYP83 in *P. lunatus*, and a CYP736 in *L. japonicus* and *T. repens*. The hydroxynitriles are glucosylated by members of the UGT85 family. The stereochemistry of the oximes produced by *P. lunatus* is presently unknown, the forms shown are the *Z*‐isomer. Colored boxes indicate that genes belong to the same gene family

All *P. lunatus* genes identified in this study were obtained from the same leaf cDNA sample, indicating that the genes are simultaneously expressed in this cyanogenic glucoside producing tissue. To establish if the genes constituted a functional biosynthetic pathway for linamarin and lotaustralin, we transiently co‐expressed various gene combinations by *Agrobacterium*‐mediated co‐infiltration of *N. benthamiana* leaves. Expression of the two possible combinations that made up a full *P. lunatus* gene set, *CYP79D71*/*CYP83E46*/*UGT85K31* and *CYP79D71*/*CYP83E47*/*UGT85K31*, both resulted in efficient production of linamarin and lotaustralin (Figure 3). The production of these compounds required the presence of all three genes. These results, therefore, suggest that a functional biosynthetic pathway for linamarin and lotaustralin in *P. lunatus* consists of CYP79D71, CYP83E46/47, and UGT85K31 (Figure 4).

This is the first report of a role for CYP83s in the biosynthesis of cyanogenic glucosides. In *L. japonicus*, *Lj*CYP736A2 is the enzyme converting the oximes to the corresponding cyanohydrins (Takos et al., 2011). To evaluate if CYP736A2‐like enzymes play a similar role in linamarin and lotaustralin biosynthesis in *P. lunatus*, we additionally cloned a full‐length CYP736 gene from leaf cDNA using degenerate primers based on the *P. vulgaris* genes most closely related to *LjCYP736A2* (Table S1). This gene was assigned the name *CYP736A222* (Nelson, 2009) and showed the highest sequence similarity (90% amino acid sequence identity) to one of the selected *P. vulgaris* genes (*Phvul.004G159600*) localized on chromosome 4. Transient expression of *PlCYP736A222* in combination with *PlCYP79D71* and *PlUGT85K31* did not result in the production of linamarin or lotaustralin, or any other notable compound (Figure S3a). Combining *PlCYP736A222* with the pathway genes from *L. japonicus*, *LjCYP79D3,* and *LjUGT85K3* also did not result in product formation (Figure S3b), demonstrating that *Pl*CYP736A222 could not substitute for *Lj*CYP736A2 from *L. japonicus*. These results support the notion that within the legumes, different cytochrome P450 families, CYP736 in *L. japonicus* and *T. repens*, and CYP83 in *P. lunatus*, have been recruited for the production of the cyanogenic glucosides linamarin and lotaustralin (Figure 4).

### 2.4 Distribution of cyanogenic glucosides within the genus *Phaseolus*

Unlike *P. lunatus*, the common bean *P. vulgaris* is not known to be cyanogenic, and reports of the presence of small amounts of linamarin in *P. vulgaris* have been questioned (Johne, 1991). However, *P. vulgaris* is able to produce the oximes derived from valine and isoleucine, and uses these as volatile defense compounds (Wei et al., 2006). The CYP83E46/47 ortholog present in the *P. vulgaris* genome accounts for an oxime‐metabolizing enzyme. Subsequent glucosylation of the resulting reactive hydroxynitrile compounds, for instance by a promiscuous UDP‐glucosyltransferase, would result in the synthesis of linamarin and lotaustralin. This suggests a plausible evolutionary pathway toward cyanogenesis, which would require the additional recruitment of an activating β‐glucosidase.

To clarify the occurrence of cyanogenesis, we tested the leaves of 60 wild (natural) and cultivated accessions of *P. vulgaris* and 71 *P. lunatus* accessions for their ability to release HCN using detection with Feigl‐Anger paper, and performed metabolite analysis of leaf extracts using LC–MS (Table S2). All of the *P. vulgaris* accessions were acyanogenic, and LC–MS analysis showed that they essentially lacked linamarin and lotaustralin. Trace amounts of linamarin seemed to be present in a few accessions (e.g., G24576, a wild type from Oaxaca, Mexico) based on the presence of a matching *m/z* value and retention time (Figure S4). However, due to the low levels, no MS2 spectrum could be obtained for a more conclusive identification of the compound. In contrast, most of the *P. lunatus* accessions contained linamarin and lotaustralin and were either HCN positive or polymorphic. Among the five *P. lunatus* accessions that were acyanogenic in our tests, a single cultivar (G26193, a landrace from Kivu, Congo) lacked both linamarin and lotaustralin. Such polymorphisms typically result from the absence of an activating β‐glucosidase or lack of cyanogenic glucoside production, and occur in natural populations as a result of balancing selection pressures (Olsen & Small, 2018).

Cyanogenesis was previously reported for *P. maculatus*, *P. marechalii*, *P. polystachios*, *P. ritensis*, *P. jaliscanus,* and *P. salicifolius*, which are all cross‐compatible with *P. lunatus* (Baudoin et al., 1991). In the phylogeny of the genus *Phaseolus*, these six species belong to the Polystachios group, the sister group to the Lunatus group named after *P. lunatus* (Delgado‐Salinas, Bibler, & Lavin, 2006). We obtained a broader overview of the occurrence of linamarin and lotaustralin within the *Phaseolus* genus by analyzing leaf extracts from a selection of wild and cultivated accessions drawn from the germplasm collection at CIAT (International Center for Tropical Agriculture) (Table 1, Table S2). The accessions were selected based on established phylogenetic relationships and represented 35 distinct species from all eight groups within the *Phaseolus* genus. Apart from *P. lunatus*, three other species belonging to the Lunatus group, *P. augusti*, *P. lignosus,* and *P. pachyrrhizoides*, were cyanogenic and clearly contained linamarin and lotaustralin. We also confirmed cyanogenesis and the presence of these two cyanogenic glucosides in members of the Polystachios group, which included the newly tested species *P. rotundatus* and *P. nodosus*. In the accessions of *P. marechalli* (G40812) and *P. nodosus* (G40899) we tested, lotaustralin was the more dominant compound.

**TABLE 1:** Species of the genus *Phaseolus* tested in this study for the occurrence of cyanogenesis and the presence of the cyanogenic glucosides linamarin and lotaustralin. The species are grouped according to the eight recognized clades within the genus (Delgado‐Salinas et al., 2006). *P. lunatus* *and P. vulgaris are indicated in bold*

Clade	Species	Cyanogenesis	Cyanogenic glucosides
Tuerckheimii	*P. chiapasanus* *P. oligospermus* *P. zimapanensis*	No No No	trace No No
Pauciflorus	*P. pluriflorus*	No	No
Pedicellatus	*P. altimontanus* *P. grayanus* *P. esperanzae* *P. pedicellatus*	No No No No	No No trace No
Filiformis	*P. angustissimus* *P. carterae* *P. filiformis*	No No No	No No No
Vulgaris	*P. acutifolius* *P. albescens* *P. coccineus* *P. costaricensis* *P. dumosus* *P. parvifolius* ***P. vulgaris***	No No No No No No **No**	No No No/trace No/trace No trace **No/trace**
Leptostachyus	*P. lepstostachyus* *P. macvaughii*	No No	No trace
Lunatus	*P. augusti* *P. lignosus* ***P. lunatus*** *P. pachyrrhizoides*	Yes Yes **Yes** Yes	Yes Yes **Yes** Yes
Polystachios	*P. maculatus* *P. marechalii* *P. nodosus* *P. polystachios* *P. rotundatus* *P. salicifolius*	Yes Yes Yes Yes Yes Yes	Yes Yes Yes Yes Yes Yes
Unassigned	*P. glabellus* *P. macrolepis* *P. magnilobatus* *P. microcarpus* *P. oaxacanus*	No No No No No	trace trace trace No No

The Vulgaris group of the genus *Phaseolus* contains several other domesticated legumes, such as *P. acutifolius* (tepary bean), *P. coccineus* (runner bean), and *P. dumosus* (year‐long bean). Cultivars and natural accessions of these three species, as well as natural accessions of *P. parvifolius*, *P. albescens*, and *P. costaricensis*, were shown to essentially contain no, or occasionally trace amounts, of the cyanogenic glucosides (Table 1, Table S2). Species in the Filiformis, Pedicellatus, Tuerckheimii, Pauciflorus, and Leptostachyus groups of the *Phaseolus* genus were represented by a single natural accession of each selected species, and exhibited a similar absence of linamarin and lotaustralin. Our data support the notion that in the genus *Phaseolus*, the occurrence of linamarin and lotaustralin as functional cyanogenic defense compounds is limited to species belonging to the closely related Polystachios and Lunatus groups.

## 3 DISCUSSION

### 3.1 Oxime‐metabolism and the repeated evolution of cyanogenesis

Cyanogenic glucosides occur widely in the plant kingdom as chemical defense compounds, and these α‐hydroxynitrile glucosides are synthesized from a selected set of amino acids, depending on the species. The synthesis of dhurrin from tyrosine in *S. bicolor* was the first biosynthetic pathway for a cyanogenic glucoside to be elucidated, revealing the role of a cytochrome P450 of the CYP79 family (CYP79A1) in the conversion of an amino acid into an oxime, and the role of a CYP71 (CYP71E1) in the production of the α‐hydroxynitrile aglycone (Bak et al., 1998; Koch et al., 1995). The conversion of an amino acid into an oxime by a member of the CYP79 family as a first step in cyanogenic glucoside biosynthesis, is observed in both gymnosperms and angiosperms (Gleadow & Møller, 2014; Luck et al., 2017). And although oxime production by members of the CYP79 family has evolutionary ancient roots, cyanogenic glucoside production and cyanogenesis are traits that we now consider to have evolved repeatedly within a number of plant lineages, which is further supported by our present findings (Takos et al., 2011).

In cassava (*M. esculenta*), Japanese apricot (*P. mume*), and almond (*P. dulcis*), the second enzymatic steps in the production of their cyanogenic glucosides, are also catalyzed by members of the CYP71 family (Jørgensen et al., 2011; Thodberg et al., 2018; Yamaguchi et al., 2014). However, work on cyanogenesis in *L. japonicus* revealed that a related gene family, CYP736, was recruited for this oxime‐metabolizing step and results indicated that cyanogenesis evolved independently in the three plant lineages leading to either *L. japonicus*, *M. esculenta* or *S. bicolor* (Takos et al., 2011). Recent work in *Eucalyptus cladocalyx*, which produces the phenylalanine‐derived cyanogenic glucoside prunasin, showed that in this species the conversion of the oxime to a hydroxynitrile involves the sequential action of not one but two cytochrome P450 enzymes, CYP706C55 and CYP71B103, providing a further example of the repeated evolution of cyanogenic glucoside biosynthesis (Hansen et al., 2018).

This report on cyanogenesis in *P. lunatus*, adds the CYP83s to the list of cytochrome P450 families involved in a cyanogenic glucoside biosynthetic pathway. Olsen and Small (2018) reported that the genes of the pathway in white clover (*T. repens*) were orthologous to the ones in *Lotus*, making the argument that cyanogenesis in these legumes was present in their common ancestor. Whereas *Tr*CYP79D15 is a clear ortholog of *Lj*CYP79D3 and *Lj*CYP79D4 from *L. japonicus*, the same cannot be said of *Pl*CYP79D71 (Figure 1b). The identification of *Pl*CYP83E46 and *Pl*CYP83E47 as oxime‐metabolizing enzymes in *P. lunatus* supports the idea that within the legumes, cyanogenesis evolved at least twice (Figures 1a and 4), with the biosynthetic pathway in *Vicia* spp. remaining to be elucidated. Therefore, this study contributes to an emerging picture of variations and flexibilities in oxime‐based biosynthetic pathways in plant specialized metabolism, of which cyanogenesis is only one possible incarnation (Sørensen, Neilson, & Møller, 2018).

For example, members of the CYP79, or the CYP71 and CYP83 families, acting on specific amino acids or their derived oximes, respectively, have been shown to function in a variety of non‐cyanogenic plant chemical defense pathways. In poplar (*Populus trichocarpa*), the enzymes CYP79D6 and CYP79D7, and CYP71B40 and CYP71B41, produced oximes and nitriles as volatile defense compounds upon herbivory (Irmisch et al., 2013, 2014). In *Arabidopsis thaliana*, CYP79B2 and CYP79B3 convert tryptophan into indole‐3‐acetaldoxime (IAOx), which is the substrate taken by CYP71A13 to produce indole‐3‐acetonitrile as an intermediate in the biosynthesis of the indole phytoalexin camalexin (Glawischnig, Hansen, Olsen, & Halkier, 2004; Nafisi et al., 2007). But IAOx is also the substrate for the oxime‐metabolizing enzyme CYP83B1, which channels it into the biosynthetic pathway for indole glucosinolates (Bak, Tax, Feldmann, Galbraith, & Feyereisen, 2001). In *Arabidopsis*, CYP79A2 is the first enzyme in the biosynthesis of phenylalanine‐derived aromatic glucosinolates, whereas CYP79F1 and CYP79F2 are involved in the biosynthesis of the various aliphatic glucosinolates that are produced from chain‐elongated methionine derivatives (Halkier & Gershenzon, 2006). CYP83A1 is the main oxime‐metabolizing enzyme in the production of aliphatic glucosinolates, whereas both CYP83A1 and CYP83B1 are involved in the biosynthesis of the aromatic glucosinolates (Naur et al., 2003).

### 3.2 From oxime‐based volatiles to cyanogenic glucosides in *Phaseolus* spp

The cytochrome P450 enzymes orthologous to *Pl*CYP79D71 and *Pl*CYP83E46/E47 in *Phaseolus* species that do not produce cyanogenic glucosides, such as *P. vulgaris*, are likely to play a related role in plant defense. In *P. vulgaris*, the release of volatile oximes in response to agromyzid flies was previously reported (Wei et al., 2006). Tissue damage by adults and the leaf mining larvae of two *Liriomyza* species resulted in the release of 2‐methylpropanal oxime, 2‐methylbutanal oxime, and 3‐methylbutanal oxime. These are the oximes produced from the amino acids valine, isoleucine, and leucine, respectively, by the action of cytochrome P450 enzymes of the CYP79 family (Figure 4). The presence of *Pl*CYP79D71 and *Pl*CYP79D72 orthologs in *P. vulgaris,* therefore, accounts for the biosynthesis of all three types of oxime volatiles observed. A report of the release of oxime and nitrile volatiles also exist for *P. lunatus* in response to jasmonic acid treatment and feeding damage by two‐spotted spider mites (*Tetranychus urticae*) (Dicke, Gols, Ludeking, & Posthumus, 1999). The oximes reported by Dicke et al. were *O*‐methylated derivates of the ones observed in *P. vulgaris*, and part of a complex blend of volatiles from different biosynthetic pathways that attracted the carnivorous mite *Phytoseiulus persimilis*. These observations support a role for *Pl*CYP79D72 in the production of leucine‐derived oximes in *P. lunatus*, and suggest that *Pl*CYP79D71 has an additional role in volatile production.

The preexistence of plant chemical defenses based on the release of volatile oximes and nitriles, would greatly facilitate the subsequent evolution of cyanogenic defense strategies. Possible evolutionary steps may include changes in gene expression, for example from insect damage‐induced CYP79 expression (Irmisch et al., 2013) to expression during early leaf development, and the recruitment of appropriate UDP‐glucosyltransferase and β‐glucosidase enzyme activities. Both of these classes of enzymes are notoriously promiscuous, given for example the involvement of UGTs in the detoxification and sequestration of reactive metabolites and xenobiotics. The traces of linamarin observed in some of the *P. lunatus* samples, could result from unspecific UDP‐glucosyltransferase activities associated with avoiding auto‐toxicity issues. Following recruitment, increased substrate specificity may evolve over time (Khersonsky & Tawfik, 2010; Lai et al., 2014).

### 3.3 Biosynthetic gene clusters and pathway evolution

The fact that the biosynthetic genes for linamarin and lotaustralin were clustered in the genome of *L. japonicus*, aided the identification of *Lj*CYP736A2 as the oxime‐metabolizing enzyme (Takos et al., 2011). Although we do not have genomic sequence data for *P. lunatus*, the initial identification of *PlCYP83E46* and *PlCYP83E47* was also based on co‐localization of *CYP79* and *CYP83* genes in the related *P. vulgaris* genome. In addition to *L. japonicus*, the presence of biosynthetic gene clusters for cyanogenic glucosides or related non‐cyanogenic β‐ and γ‐hydroxynitrile glucosides has now been reported in cassava, sorghum, white clover, and barley (Ehlert et al., 2019; Knoch et al., 2016; Olsen & Small, 2018; Takos et al., 2011).

We have proposed that gene clustering results from and promotes the co‐inheritance of favorable combinations of alleles that are under balancing selection pressures, as is the case for the biosynthetic genes of chemical defense pathways that provide a conditional advantage (Takos & Rook, 2012). White clover is a well‐studied example of a species in which such an adaptive polymorphism (presence/absence) of cyanogenesis occurs in natural populations by either lack of cyanogenic glucoside production or the absence of an activating β‐glucosidase. Interestingly, Olsen and Small (2018) observed that in *T. repens* the adaptive polymorphism that involves loss of cyanogenic glucoside biosynthesis occurs through presence or absence of the complete gene cluster. Similarly, barley cultivars that lacked hydroxynitrile glucoside production, contained a deletion of the central part of the gene cluster (Ehlert et al., 2019). The genomic organization in a biosynthetic gene cluster is not an inherent trait of the cyanogenic glucoside biosynthetic pathway as it is not observed in almonds (Thodberg et al., 2018) or sugar gum (Hansen et al., 2018). Individuals of these two perennial species are likely to experience seasons with high herbivore pressure during their lifetime and consequently cyanogenesis is more of a constitutively beneficial trait. Our present analysis of cyanogenesis and cyanogenic glucosides in *P. lunatus* (Table S2), shows the characteristic presence/absence polymorphisms that are observed in other annual legumes, reflecting the balancing selection pressures necessary to promote gene cluster formation (Takos & Rook, 2012).

Once a gene cluster is established, the resulting co‐inheritance could greatly facilitate co‐evolution of the interacting genes and support the formation of alternative biosynthetic routes following local gene duplications and functional divergence. This is observed for a number of biosynthetic gene clusters in plant specialized metabolism. The gene cluster for cyanogenic glucosides in *L. japonicus* has gained the ability to produce a set of alternative, non‐cyanogenic β‐ and γ‐hydroxynitrile glucosides called rhodiocyanosides, with the pathways diverging at the oxime metabolizing step (Takos et al., 2011). This ability to additionally produce rhodiocyanosides is restricted to a single clade within the *Lotus* genus (Lai et al., 2014). Similarly, the biosynthetic gene cluster in barley contains several members of the CYP79, CYP71 and UGT85 gene families, coordinately producing a mixture of five leucine‐derived hydroxynitrile glucosides (Ehlert et al., 2019; Knoch et al., 2016).

A genomic, phylogenetic, and biochemical comparison in several species of the genus *Solanum* was used to describe the evolution of a terpene biosynthetic gene cluster and its various products, which involved gene duplication, gene conversion, pseudogenization, and the functional divergence of terpene synthases (Matsuba et al., 2013). Similarly, gene duplication without translocation and functional divergence played an important role in the evolution of two diterpenoid gene clusters in the genus *Oryza* (Miyamoto et al., 2016; Swaminathan, Morrone, Wang, Fulton, & Peters, 2009). Our results in *Phaseolus* suggest that a small clustered pathway for oxime‐based metabolism, as present in *P. vulgaris*, has evolved to produce cyanogenic glucosides. This is supported by the restricted occurrence of cyanogenesis to the Lunatus and Polystachyus clades of the genus, whereas the occurrence in *P. lunatus* of two paralogous CYP83 genes, *PlCYP83E46* and *PlCYP83E47*, may have resulted from a gene duplication event. The future availability of genome sequences of additional *Phaseolus* species will provide further insights in the genomic organization of oxime‐based chemical defense pathways and their evolution in this genus.

## 4 MATERIALS AND METHODS

### 4.1 Plant materials and growth conditions

*Phaseolus lunatus* accession “Hopi Lima” (PHAS8445) was obtained from the Leibniz Institute for Plant Genetics and Crop Plant Research (IPK) seed collection in Gatersleben, Germany. The seeds were germinated on wet cotton, seedlings transferred to soil, and grown under greenhouse conditions in Copenhagen, Denmark. Similarly, *Nicotiana benthamiana* plants were germinated from seed and grown in soil. *Phaseolus* spp. cultivars and accessions selected from the CIAT germplasm collection were grown in the institute's greenhouses in Cali, Colombia.

### 4.2 RNA extraction, cDNA synthesis, and gene cloning

RNA was prepared from *P. lunatus* leaves accession “Hopi Lima” (100 mg) using a RNeasy plant mini kit with on‐column DNase I digestion (Qiagen). First‐strand cDNA was synthesized from 2.5 μg of total RNA using SuperScript III reverse transcriptase (Invitrogen) in a reaction primed with 50 μM oligo (dT)_20_. PCR products of gene fragments were obtained using Hotmaster Taq DNA polymerase, gel purified and cloned into the pDrive Cloning Vector (Qiagen). 5′ and 3′ RACE PCR were performed using the FirstChoice™ RLM‐RACE Kit (Ambion). cDNA clones of complete coding regions were obtained using Phusion High‐Fidelity DNA Polymerase, gel purified, and cloned by Gateway recombination reaction into the entry vector pDONOR207 (Invitrogen).

### 4.3 Transient expression in leaves of *N. benthamiana* plants

Expression constructs containing cDNAs of *CYP79D71*, *CYP79D72*, *CYP83E46*, *CYP83E47*, *CYP736A222*, and *UGT85K31* from *P. lunatus* under control of the CaMV 35S promoter, were generated by cloning into the pJAM1502 vector, and transforming the plasmids to *A. tumefaciens* strain AGL1 by electroporation, as previously described (Takos et al., 2011). Constructs for the *L. japonicus* genes *CYP79D3*, *CYP736A2,* and *UGT85K3* in pJAM1502, and transient expression in *N. benthamiana* leaves by co‐infiltration of selected cultures are as described in Takos et al. (2011). After 4 days, leaf disks of 1 cm diameter were cut from the infiltrated leaves and extracted in 85% (v/v) methanol for metabolite analysis by LC–MS.

### 4.4 Detection of cyanogenesis with Feigl‐Anger paper

Cyanogenesis was visualized using Feigl‐Anger paper, which was prepared as described in Takos et al. (2010). Plant tissue was disrupted by grinding in 300 µl of 20 mM MES buffer, pH 6.5 in 96‐well plates and exposed to Feigl‐Anger paper. After incubation for 10–30 min at room temperature, the paper was removed and HCN release was detected by the development of blue color.

### 4.5 LC–MS analysis

For metabolite profiling of hydroxynitrile glucosides, plant material was extracted by boiling in 85% methanol, essentially as described in Takos et al. (2011). Samples of extracts prepared at CIAT were dried down in 96‐wells microtiter plates and shipped to Copenhagen for analysis, where they were redissolved in 85% methanol (v/v) and filtered prior to analysis. Analytical LC–MS was performed using an Agilent 1100 Series LC (Agilent Technologies) coupled to a Bruker HCT‐Ultra ion trap mass spectrometer (Bruker Daltonics). A Zorbax SB‐C18 column (Agilent, 2.1 mm × 50 mm, 1.8 μM) was used with chromatography conditions as described previously (Takos et al., 2010). Compounds were localized in extracted ion chromatograms as sodium adduct ions: linamarin (*m/z* 270) and lotaustralin (*m/z* 284).

### 4.6 Phylogenetic analysis

Homologous protein sequences were obtained by database searches using blastp at NCBI (www.ncbi.nlm.nih.gov), Phytozome (phytozome.jgi.doe.gov), and from the *L. japonicus* genome sequence available at the Kazusa DNA Research Institute (www.kazusa.or.jp/lotus/index.html). Chloroplast matK sequences were obtained from NCBI by text search for the various legume species. Amino acid sequences were aligned using the MUSCLE algorithm, followed by an analysis of phylogeny in MEGA version X (Kumar, Stecher, Li, Knyaz, & Tamura, 2018).

### 4.7 Accession numbers

Sequence data of the *P. lunatus* genes identified in this study have been assigned the following accession numbers: *CYP79D71* (LR699072), *CYP79D72* (LR699067), *CYP83E46* (LR699068), *CYP83E47* (LR699069), *CYP736A222* (LR699070), *UGT85K31* (LR699071).

## CONFLICT OF INTEREST

The authors declare no conflict of interest associated with the work described in this manuscript.

## AUTHOR CONTRIBUTIONS

D.L., A.M., B.R., D.D., and F.R. designed the study. D.L. and A.M. cloned genes with assistance from B.D. and F.R. D.L. and A.M. performed expression studies. E.M., C.O., and G.C. grew *Phaseolus* accessions, tested for cyanogenesis and prepared extracts at CIAT. C.E.O. performed LC–MS and M.P. provided additional metabolite analysis. A.M., B.L.M., D.L., and F.R. wrote the manuscript with contributions from the other authors.
