# Supplementary material for: Biosynthesis of cyanogenic glucosides in Phaseolus lunatus and the evolution of oxime‐based defenses
Source: Plant Direct. 2020 Aug 4;4(8):e00244. doi: 10.1002/pld3.244 (PMC7402084; doi:10.1002/pld3.244)
Supplement: Supplementary file 1 [file PLD3-4-e00244-s001.pdf]

SUPPLEMENTARY DATA

WITH

Biosynthesis of Cyanogenic Glucosides in *Phaseolus lunatus* and the Evolution of Oxime-based Defenses

Daniela Lai, Alexandra B. Maimann, Eliana Macea, César H. Ocampo, Gustavo Cardona, Martina Pičmanová, Behrooz Darbani, Carl Erik Olsen, Daniel Debouck, Bodo Raatz, Birger Lindberg Møller and Fred Rook

Supplemental Table S1: oligonucleotide primers used in this study.

Degenerate primers for gene cloning.
Primers are designed based on conserved amino acid motifs distinguishing the different CYP gene families in legumes species. DNA sequences from homologous genes from *L. japonicus*, *T. repens* and *P. vulgaris* guided nucleotide selection to account for legume specific codon preference.

	**Forward**	**Reverse**
CYP79<br>(use in 4 combinations possible)	5’-GGYAAYCTYCCTGARATGCTTGCAAA-3’<br>5’-TGAACACYSAAATWGCNTGTATYCGCCTA-3’	5’-AGCAACTCAGGYTGGTTTAWCATYTCTGC-3’<br>5’-CKAGCAAAYAASATYAYWGTCATTGTKGT-3’
CYP736	5’-CTYCACATGYTAGGRAAACTMCCACA-3’	5’-TAACATCTTCTCTACACTCRCGAGGTAC-3’
CYP83	5’-CAMAGAAATAYGGNYCTWTATTTTCMCTT-3’	5’-TRTTGTCTTKGCTGGWATTTCRTAARC-3’
UGT85	5’-GTCYCWTGGASMAGAMTTTGTWAAGGG-3’	5’-GAARCCYYTKTCTTTMTCRGGAAAATG-3’

Gene specific primers used in 5’ and 3’ RACE procedures
Additional (linker) primers are part of the FirstChoice™ RLM-RACE Kit (Ambion).
Primers are used in consecutive PCR reactions (nested PCR).

	**5’- RACE**	**3’-RACE**
CYP79D71	5’-CACCAAAATGGTGGTTATATATCCTCT-3’<br>5’-ATGAGATCATTGCACGGGGTTAGTG-3’	5’-CAGTGGACAATCCATCAAATGCTT-3’<br>5’-GCTTTTGAATGGGCACTAGGTG-3’
CYP79D72	5’-ACAAGGGCTGTGGTGAGATATCCA-3’<br>5’-ACAAGAGAAGTGGTTATGCTTGTGG-3’	5’-GGTGGATAATCCATCAAATGCAG-3’<br>5’-GCAGTTGAATGGGGTCTGGCAGA-3’
CYP736A222	5’-TATGGCTGGGACCTGCCCCAGCTTT-3’<br>5’-GGTTCGATGTGGGAGTTTTCCTAG-3’	5’-AAACATGTGGAGGAAAATGACTTG-3’<br>5’-AACACTAAGGTTGCACCCGGCTGG-3’
CYP83E46	5’-TCTCTAACCCATTGTAGGACAATT-3’<br>5’-TTTAGGTGAGGAGATGACAATGGA-3’	5’-GTAAGAAAGAGTTTCTAGAAGAAG-3’<br>5’-GAGACTGTATCTACCAGCACCATT-3’
CYP83E47	5’-TATCTAACCCATCATAAGACAATC-3’<br>5’-TTTAGCTGAGGAGACGACAATGGC-3’	5’-GTAAGAAAGAATTTCTAGAAGATC-3’<br>5’-CAGACTGTATCCAGCAGCACCAAC-3’
UGT85K31	5’-GGTATACCCAAATCCTTCGCAACTTT-3’<br>5’-CAATTATGCAACTAATTGGAGGCACG-3’	5’-ATAAACACATTCGAAGACTTGGAGGG-3’<br>5’-TACCATTGGTCCATTTGACTTGCTTG-3’

Gateway cloning PCR primers
The primers additionally contained the attB1 and attB2 Gateway cloning sites: ggggacaagtttgtacaaaaaagcaggct and ggggaccactttgtacaagaaagctgggt. The gene specific sequences (excluding attB1 and attB2 sites) were:

	**Forward**	**Reverse**
CYP79-A	5’-ATGGAAGATGCTCGTTCTC-3’	5’-TTAAAGGTGATATAAATGT-3’
CYP79-B	5’-ATGGTACAGTATTCTATCT-3’	5’-TCACCCAATATAAAGTTGT-3’
CYP736A222	5’-ATGTTGCCTCAAGAAG-3’	5’- CTAACTAGCTAATCGG -3’
CYP83-A	5’-ATGGTGTCACCACTTCTGA-3’	5’-TTAGATTAGATTTAGATTC-3’
UGT85	5’-ATGGACTCCCTTCAAAGCT-3’	5’-TCAAATAGCCTTGTGGTTT-3’

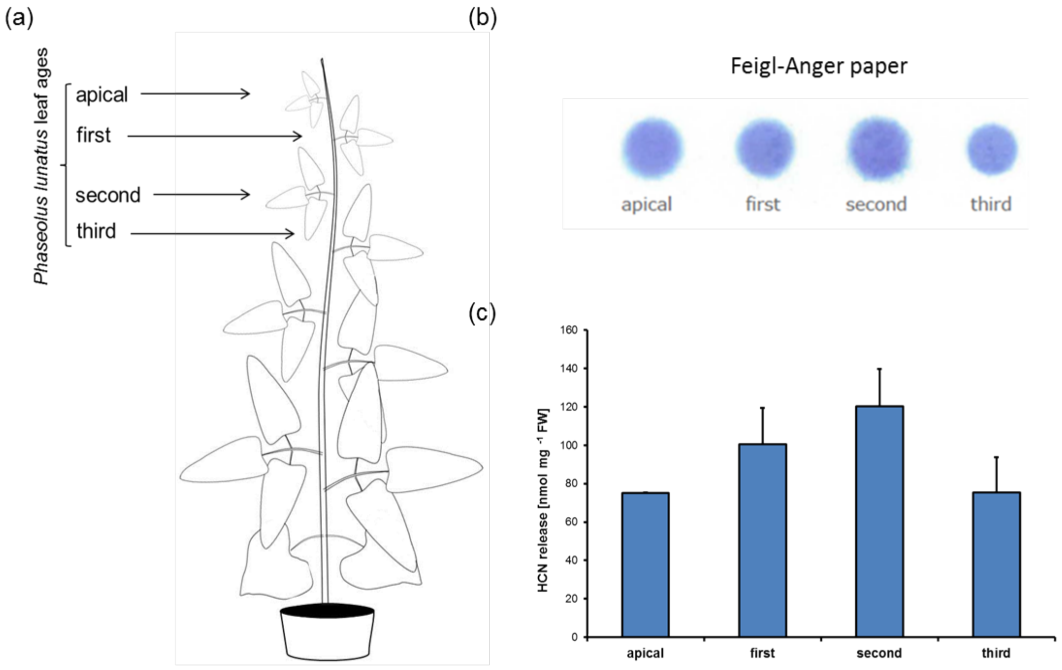

Supplemental Figure S1. Cyanogenic potential in young leaves of *P. lunatus*.
(a) *P. lunatus* plant overview of used leaf ages
(b) Semi-quantitative analysis of HCN release by using HCN-sensitive Feigl-Anger paper
(c) Quantitative analysis of HCN release in the apical, first, second and third leaf of *P. lunatus*. Values are the mean of three technical and three biological replicates ± SE.

Supplemental method:

Quantitative HCN detection by using a colorimetric assay

The cyanogenic potential of *P. lunatus* leaves was determined based on the calorimetric method described by Lambert *et al*. (1975) as modified by Halkier & Møller (1989). A small plant sample (5–10 mg) was added to 200 µl MES buffer (20 mM, pH 6.5), freeze/thawed three times on ice, and incubated at 30ºC for 30 min. The incubation was stopped by freezing the samples in liquid nitrogen and addition of NaOH (40 µl, 6 M) to the frozen samples. After thawing up the samples at RT, the hydrogen cyanide potential was determined by addition of glacial HOAc (12.5 µl) immediately followed by 50 µl reagent A (50 mg succinimide and 125 mg *N*-chlorosuccinimide in 50 ml water) and 50 µl reagent B (3 g barbituric acid and 15 ml pyridine in 35 ml water. After thorough mixing and incubation (5 min), the hydrogen cyanide content was determined spectrophotometrically (450-nm to 700-nm scan; UV/VIS spectrometer, Lambda 800; Perkin-Elmer).

Lambert *et al. Anal. Chem.* **47,** 916–918 (1975).
Halkier, B. a & Møller, B. L. *Plant Physiol.* **90,** 1552–1559 (1989).

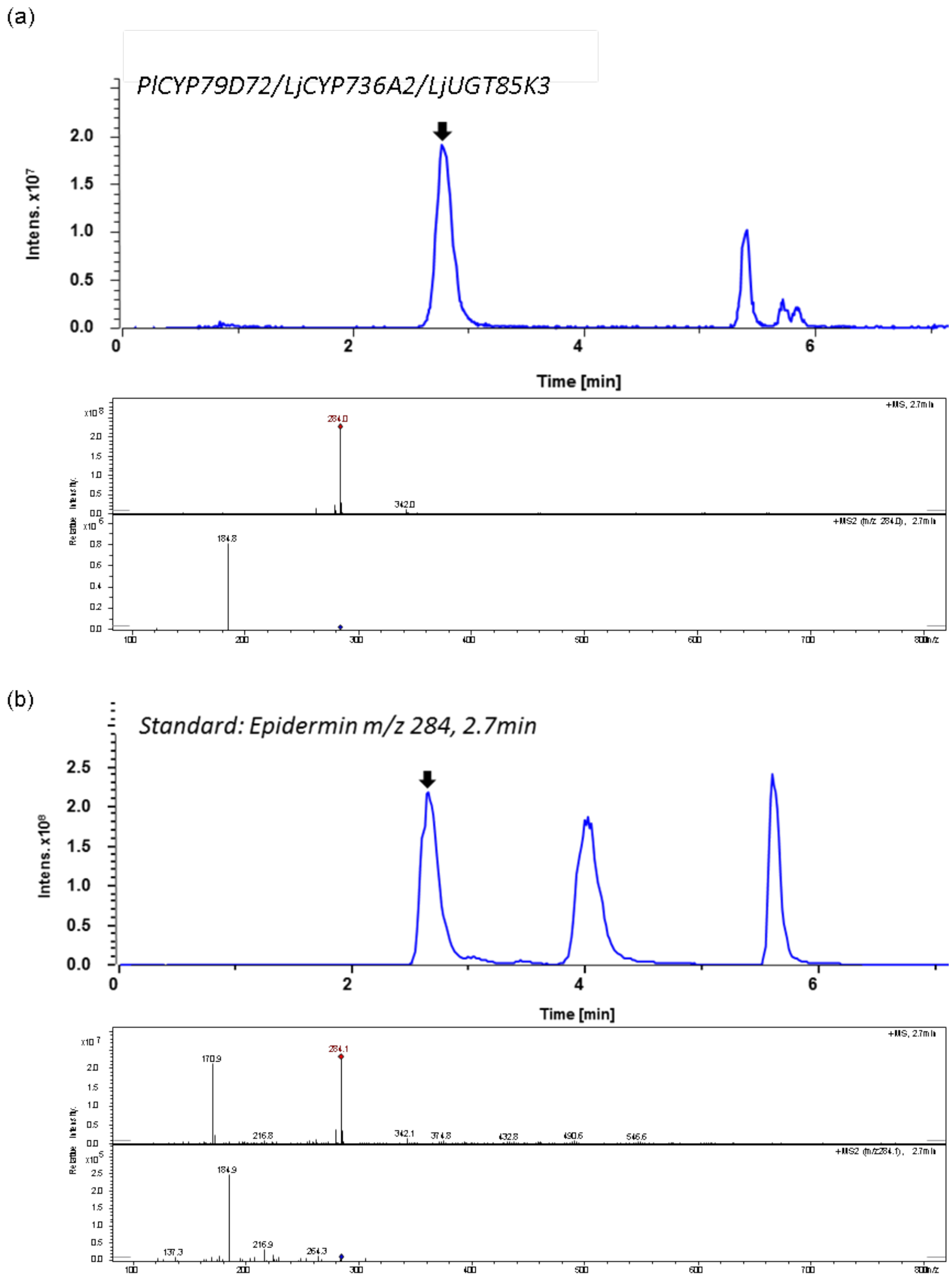

Supplemental Figure S2: *Pl*CYP79D72 has leucine as its substrate and in combination with *Lj*CYP736A2 and *Lj*UGT85K3 produces the β-hydroxynitrile glucoside epidermin. (a) Extracted ion chromatogram of tobacco leaves co-infiltrated with the *P. lunatus* gene *CYP79D72* and the *L. japonicus* genes *CYP736A2* and *UGT85K3*. Extracted ion peaks are for sodium adducts: epidermin (m/z 284, RT 2.7 min.) and lotaustralin (m/z 284, RT 5.5 min.). (b) Extracted ion chromatogram of an *Hordeum vulgare* leaf extract. Extracted ion peaks are for sodium adducts: epidermin (m/z 284, RT 2.7 min.), dihydroosmaronin (m/z 284, RT 4.1 min.) and epiheterodendrin (m/z 284, RT 5.6 min.). Mass spectra of the m/z 284 ion and its fragmentation (MS2) are for the epidermin peak (indicated with arrow).

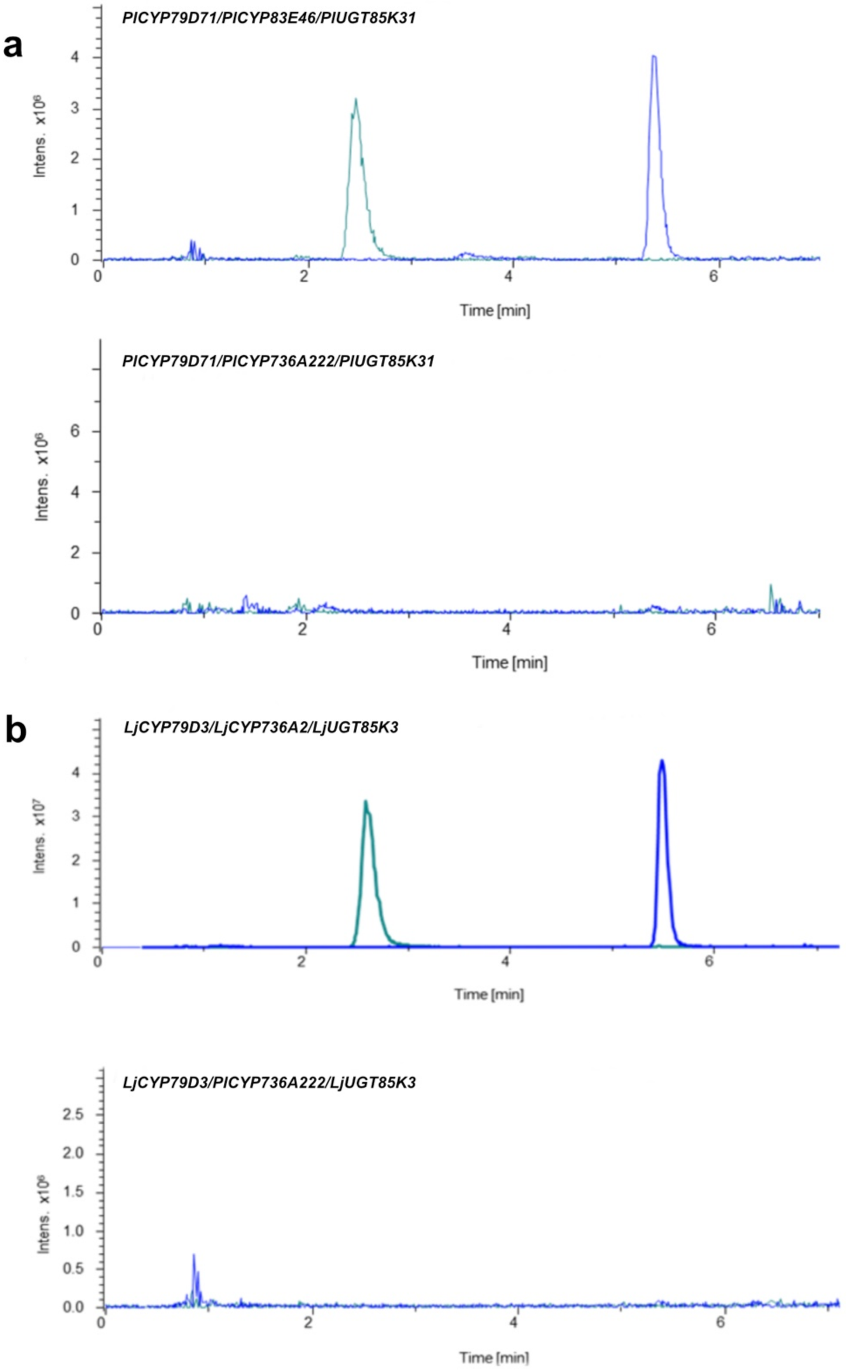

Supplemental Figure S3: CYP736A222 does not substitute for CYP83E46 or CYP736A2 in the production of linamarin and lotaustralin.

Extracted ion chromatogram of tobacco leaves co-infiltrated with (a) the indicated *P. lunatus* gene combinations, or (b) the *L. japonicus* biosynthetic genes in combination with *PlCYP736A222*. Extracted ion peaks are for sodium adducts: linamarin (m/z 270, cyan), lotaustralin (m/z 284, blue).

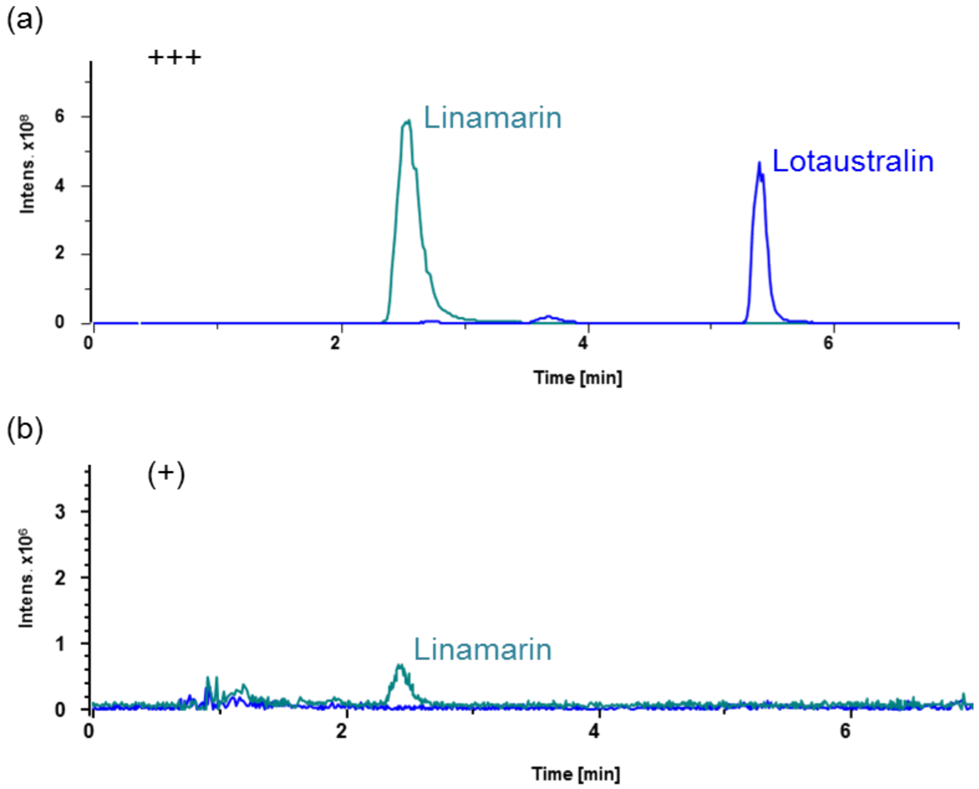

Supplemental Figure S4: **Examples of differences in cyanogenic glucosides levels in *Phaseolus spp.* accessions.**

Extracted ion chromatogram of two *Phaseolus spp.* accessions with high levels of cyanogenic glucosides in (a) and only trace amounts in (b). Note the 100x difference in the scaling of signal intensity (10^6 versus 10^8). Extracted ion peaks are for sodium adducts: linamarin (m/z 270, cyan) and lotaustralin (m/z 284, blue).

Supplemental Table S2. ***Phaseolus spp.*** **natural and cultivated accessions selected from the CIAT germplasm collection and tested for cyanogenesis and the presence of the cyanogenic glucosides linamarin and lotaustralin in leaf extracts.**

[1)] Cyanogenesis was determined by HCN detection with Feigl-Anger paper. An accession was considered polymorphic (or segregating) if separate individual plants showed a different response.
[2)] Relative signal strength in extracted ion chromatograms of cyanogenic glucosides: - = not detectable; (+) = possible trace amount; + = detectable; ++ = clearly present; +++ = abundant.
[3)] Countries of origin: ARG (Argentina), AZE (Azerbaijan), BDI (Burundi), BMU (Bermuda), BOL (Bolivia), BRZ (Brazil), CHL (Chile), CHN (China), CMR (Cameroon), COD (Democratic Republic of the Congo), COL (Colombia), CRI (Costa Rica), CUB (Cuba), DOM (Dominican Republic), ECU (Ecuador), ESP (Spain), ETH (Ethiopia), FRA (France), GHA (Ghana), GTM (Guatemala), HND (Honduras), HTI (Haiti), IND (India), ITA (Italy), JAM (Jamaica), LBR (Liberia), MEX (Mexico), MMR (Myanmar), MWI (Malawi), NGA (Nigeria), NLD (Netherlands), PER (Peru), PHL (Philippines), PRI (Puerto Rico), RWA (Rwanda), SLV (El Salvador), TUR (Turkey), TZA (United Republic of Tanzania), USA (United States of America), ZWB (Zambia).

Species name	CIAT Accession Nr.	Cyanogenesis[1]	Cyanogenic glucosides[2]		Country[3]	Type
			Linamarin	Lotaustralin		
P. acutifolius	G40175	negative	-	-	USA	cultivated
	G40111	negative	-	-	MEX	cultivated
	G40254	negative	-	-	MEX	natural
	G40080	negative	-	-	MEX	natural
	G40163	negative	-	-	MEX	cultivated
	G40299	negative	-	-	GTM	cultivated
	G40283	negative	-	-	USA	cultivated
	G40027	negative	-	-	CRI	cultivated
	G40206	negative	-	-	MEX	natural
P. albescens	G40897	negative	-	-	MEX	natural
P. altimontanus	G40568	negative	-	-	MEX	natural
P. angustissimus	G40685	negative	-	-	USA	natural
P. augusti	G40729	polymorphic	++	++	PER	natural

	**G40716**	positive	**++**	**+**	ARG	natural
	**G40759**	positive	**+++**	**++**	BOL	natural
P. carterae	**G40675**	negative	**-**	**-**	MEX	natural
P. chiapasanus	**G40676**	negative	**(+)**	**-**	MEX	natural
P. coccineus	**G35055**	negative	**+**	**-**	MEX	cultivated
	**G36136**	negative	**-**	**-**	MEX	natural
	**G35876**	negative	**-**	**-**	GTM	natural
	**G35205**	negative	**-**	**-**	GTM	cultivated
P. costaricensis	**G40807**	negative	**-**	**-**	CRI	natural
	**G40853**	negative	**-**	**-**	CRI	natural
	**G40894**	negative	**+**	**(+)**	CRI	natural
P. dumosus	**G35631**	negative	**-**	**-**	GTM	cultivated
	**G35314**	negative	**-**	**-**	COL	cultivated
	**G35550**	negative	**-**	**-**	MEX	cultivated
	**G35855**	negative	**-**	**-**	GTM	natural
	**G35584**	negative	**-**	**-**	GTM	cultivated
P. esperanzae	**G40817**	negative	**(+)**	**-**	MEX	natural
P. filiformis	**G40512**	negative	**-**	**-**	USA	natural
P. glabellus	**G40797**	negative	**(+)**	**-**	MEX	natural
P. grayanus	**G40517**	negative	**-**	**-**	MEX	natural
P. lepstostachyus	**G40620**	negative	**-**	**-**	MEX	natural
P. lignosus	**G40876**	positive	**+++**	**++**	BMU	natural
P. lunatus	**G26191**	polymorphic	**++**	**+**	NGA	cultivated
	**G25253**	polymorphic	**++**	**++**	CMR	cultivated
	**G26193**	negative	**-**	**-**	COD	cultivated
	**G25694**	polymorphic	**+++**	**+++**	ETH	cultivated
	**G26195**	polymorphic	**++**	**++**	RWA	cultivated
	**G25237**	polymorphic	**++**	**+**	IND	cultivated

	**G27616**	positive	+++	++	IND	cultivated
	**G25247D**	positive	+++	++	GHA	cultivated
	**G27209**	positive	+++	++	LBR	cultivated
	**G25969**	polymorphic	++	+	CHN	cultivated
	**G25820**	positive	+++	++	PHL	cultivated
	**G25625**	polymorphic	+++	++	USA	cultivated
	**G25799**	positive	+++	++	MEX	cultivated
	**G26534**	polymorphic	+++	++	MEX	cultivated
	**G27399**	polymorphic	+++	++	MEX	cultivated
	**G25975**	positive	+++	++	GTM	cultivated
	**G25295**	polymorphic	+++	++	CUB	cultivated
	**G27553**	positive	+++	++	DOM	cultivated
	**G25616**	positive	+++	++	HTI	cultivated
	**G25869**	polymorphic	+++	++	COL	cultivated
	**G27309**	positive	+++	++	ECU	cultivated
	**G27416**	positive	+++	+++	PER	cultivated
	**G25990**	polymorphic	+++	++	BOL	cultivated
	**G25785**	positive	++	+	MEX	natural
	**G26517**	positive	+++	++	MEX	natural
	**G26360**	positive	+++	++	MEX	natural
	**G26681**	positive	++	++	GTM	natural
	**G26629**	polymorphic	+	-	HND	natural
	**G25294D**	positive	+++	+++	CUB	natural
	**G27339**	positive	++	+	PRI	natural
	**G27609**	positive	++	+	SLV	natural
	**G26606**	polymorphic	+++	++	ECU	natural
	**G26348**	positive	++	+	PER	natural
	**G26547**	positive	+++	+	PER	natural

	**G25392C**	positive	++	+	CRI	cultivated
	**G25294B**	positive	++	+	CUB	cultivated
	**G26164**	polymorphic	++	+	MEX	cultivated
	**G26221**	positive	+++	++	USA	cultivated
	**G25872J**	positive	++	+	COL	cultivated
	**G25372A**	positive	++	+	SLV	cultivated
	**G25007**	polymorphic	+++	++	BRZ	cultivated
	**G27580A**	positive	+++	++	ZWB	cultivated
	**G25697**	positive	++	+	ECU	cultivated
	**G25219**	positive	+	(+)	MEX	natural
	**G25541C**	positive	++	+	ARG	cultivated
	**G26615**	positive	+++	++	COL	natural
	**G25575B**	positive	+++	++	PER	cultivated
	**G25713A**	positive	+++	++	MEX	natural
	**G25695**	positive	+++	++	PHL	natural
	**G27519**	positive	+	-	HTI	cultivated
	**G26468**	polymorphic	++	+	ECU	natural
	**G25299**	positive	++	+	IND	cultivated
	**G26533**	negative	++	+	MEX	natural
	**G25255A**	polymorphic	+++	++	ETH	cultivated
	**G26459**	polymorphic	++	+	ECU	natural
	**G26653**	negative	++	+	GTM	natural
	**G25246A**	positive	++	+	GHA	cultivated
	**G25915**	polymorphic	+++	+	PER	natural
	**G27414**	positive	++	+	MEX	natural
	**G26767**	positive	++	+	LBR	cultivated
	**G27573**	polymorphic	++	+	GTM	natural
	**G25578**	positive	++	+	BOL	cultivated

	**G27465**	positive	**+++**	**+**	MMR	cultivated
	**G25913**	polymorphic	**++**	**+**	PER	natural
	**G25239**	polymorphic	**++**	**+**	GTM	cultivated
	**G27574**	polymorphic	**++**	**+**	AZE	cultivated
	**G25388A**	positive	**++**	**+**	CRI	natural
	**G25540**	positive	**++**	**+**	ARG	cultivated
	**G26805**	polymorphic	**++**	**+**	NGA	cultivated
	**G26749**	negative	**+**	**+**	ARG	natural
	**G25315**	negative	**+++**	**++**	JAM	cultivated
P. macrolepis	**G40863**	negative	**(+)**	**-**	GTM	natural
P. maculatus	**G40809**	positive	**++**	**+**	MEX	natural
P. macvaughii	**G40760**	negative	**-**	**(+)**	MEX	natural
P. magnilobatus	**G40514**	negative	**(+)**	**-**	MEX	natural
P. marechalii	**G40812**	positive	**+**	**++**	MEX	natural
P. microcarpus	**G40538**	negative	**-**	**-**	MEX	natural
P. nodosus	**G40899**	positive	**+**	**++**	MEX	natural
P. oaxacanus	**G40861**	negative	**-**	**-**	MEX	natural
P. oligospermus	**G40595**	negative	**-**	**-**	CRI	natural
P. pachyrrhizoides	**G40770**	positive	**+++**	**++**	PER	natural
P. parvifolius	**G40185**	negative	**+**	**-**	GTM	natural
P. pedicellatus	**G40589**	negative	**-**	**-**	MEX	natural
P. polystachios	**G40783**	positive	**+**	**+**	USA	natural
P. pluriflorus	**G40532**	negative	**-**	**-**	MEX	natural
P. rotundatus	**G40728**	positive	**++**	**++**	MEX	natural
P. salicifolius	**G40860**	positive	**+**	**+**	MEX	natural
P. talamancenis	**G40814**	negative	**-**	**-**	CRI	natural
P. vulgaris	**G11032A**	negative	**-**	**-**	MEX	natural
	**G12866A**	negative	**(+)**	**-**	MEX	natural

	**G12882B**	negative	-	-	MEX	natural
	**G24576**	negative	**(+)**	-	MEX	natural
	**G24323**	negative	-	-	MEX	natural
	**G50505A**	negative	-	-	GTM	natural
	**G23436**	negative	-	-	GTM	natural
	**G51062**	negative	-	-	CRI	natural
	**G24798**	negative	-	-	COL	natural
	**G23582**	negative	-	-	ECU	natural
	**G23583**	negative	-	-	PER	natural
	**G19889**	negative	-	-	ARG	natural
	**G16392**	negative	-	-	USA	cultivated
	**G11108**	negative	-	-	MEX	cultivated
	**G9712**	negative	-	-	MEX	cultivated
	**G6992**	negative	-	-	MEX	cultivated
	**G79**	negative	-	-	SLV	cultivated
	**G23980**	negative	-	-	COL	cultivated
	**G18324**	negative	-	-	PER	cultivated
	**G12031**	negative	-	-	PER	cultivated
	**G23769D**	negative	-	-	PER	cultivated
	**G50468C**	negative	**(+)**	-	BOL	cultivated
	**G18355**	negative	-	-	CHL	cultivated
	**G13942**	negative	-	-	ARG	cultivated
	**G1477**	negative	-	-	ESP	cultivated
	**G8013**	negative	-	-	FRA	cultivated
	**G18048**	negative	-	-	NLD	cultivated
	**G3815**	negative	**(+)**	-	ITA	cultivated
	**G15568**	negative	-	-	TUR	cultivated
	**G766**	negative	-	-	ETH	cultivated

	**G19151A**	negative	-	-	TZA	cultivated
	**G22534**	negative	-	-	BDI	cultivated
	**G15366**	negative	-	-	MWI	cultivated
	**G467**	negative	-	-	IND	cultivated
	**G24279**	negative	-	-	CHN	cultivated
	**G24317**	negative	-	-	CHN	cultivated
	**G23470**	negative	-	-	MEX	natural
	**G10655**	negative	-	-	GTM	cultivated
	**G21117**	negative	-	-	COL	natural
	**G23617A**	negative	-	-	BOL	cultivated
	**G6386**	negative	-	-	MEX	natural
	**G2756**	negative	-	-	HND	cultivated
	**G23725**	negative	-	-	ECU	natural
	**G21044A**	negative	-	-	ARG	cultivated
	**G24382**	negative	-	-	MEX	natural
	**G51648**	negative	-	-	CRI	cultivated
	**G23421**	negative	-	-	PER	natural
	**G4830**	negative	-	-	BRZ	cultivated
	**G12855A**	negative	-	-	GTM	natural
	**G51281**	negative	-	-	COL	cultivated
	**G23458**	negative	-	-	PER	natural
	**G5818**	negative	-	-	CHL	cultivated
	**G23478**	negative	-	-	GTM	natural
	**G23445B**	negative	-	-	BOL	natural
	**G23416**	negative	+	-	CRI	natural
	**G19898**	negative	-	-	ARG	natural
	**G19918**	negative	-	-	MEX	cultivated
	**G12361**	negative	-	-	ECU	cultivated

	**G2433**	negative	-	-	MEX	cultivated
	**G12120**	negative	-	-	PER	cultivated
P. zimapanensis	**G40556**	negative	-	-	MEX	natural
